# Short-term outcomes of shoulder arthroplasty using the Comprehensive Convertible Glenoid system

**DOI:** 10.1016/j.jsea.2026.100101

**Published:** 2026-09-30

**Authors:** Carl M. Cirino, Joel R. Peterson, Matthew S. Fury, Jennifer A. Estrada, Peter J. Chabot, Anthony Finocchiaro, Joshua I. Mathew, Anna B. Williams, Lindsay Schuster, Michael C. Fu, Samuel A. Taylor, Joshua S. Dines, David M. Dines, Russell F. Warren, Lawrence V. Gulotta

**Affiliations:** Sports Medicine Institute, Hospital for Special Surgery, New York, NY, USA

**Keywords:** Shoulder arthroplasty, Glenoid, Metal-backed glenoid, Outcomes, Shoulder arthritis, Anatomic, American Shoulder and Elbow Surgeons (ASES)

## Abstract

**Background:**

Glenoid loosening remains the most common cause of aseptic failure following total shoulder arthroplasty. Modern metal-backed glenoid (MBG) designs with bony ingrowth and screw fixation have been developed to address this, with promising short-term results, but data remain limited.

**Methods:**

This is a retrospective review of prospectively collected data investigating the short-term outcomes of anatomic total shoulder arthroplasty (aTSA) in patients who received a MBG component in conjunction with their total shoulder arthroplasty at a single center with 4 surgeons specializing in shoulder arthroplasty. Patients who underwent aTSA with a modern MBG with a minimum of 2-year follow-up were identified, and data were collected regarding clinical, radiographic, and patient-reported outcomes.

**Results:**

Twenty-eight patients (mean age 58.0 ± 6.5 years, mean follow-up 3.8 ± 1.3 years) met inclusion criteria. Range of motion improved in all measurements. American Shoulder and Elbow Surgeons improved 47.4 points (*P* < .001), Single Assessment Numeric Evaluation increased from 28.8 to 79.0 (*P* < .001), Shoulder Activity Scale from 10.0 to 13.0 (*P* = .008), Patient-Reported Outcomes Measurement Information System increased 17.8 (*P* < .001), and visual analog scale decreased from 5.2 to 2.6 (*P* < .001). Three patients underwent conversion to reverse shoulder arthroplasty; no cases of component loosening, polyethylene dissociation, or periprosthetic fracture were observed.

**Discussion:**

The Comprehensive Convertible Glenoid system in aTSA demonstrates strong radiographic and clinical outcomes at short-term follow-up. This information can be useful in counseling patients regarding the results of this procedure. Long-term studies are needed to further elucidate the longevity of MBGs.

Modern anatomic total shoulder arthroplasty (aTSA) is a reliable and effective option in the treatment of end-stage primary glenohumeral osteoarthritis and has been associated with significantly improved pain, function, and patient satisfaction.^10^, ^15^, ^22^ The incidence of primary and revision shoulder arthroplasties being performed in the United States is dramatically increasing annually, highlighting a need for implants with not only maximal longevity in the primary setting but also minimal morbidity in the revision setting.^14^, ^25^ Despite implant design improvements, glenoid component loosening remains the most common cause for revision following aTSA, and glenoid bone loss during revision remains a significant concern.^7^, ^23^

Cemented all-polyethylene glenoid (PEG) components have traditionally been the gold standard used in aTSA; however, symptomatic loosening and revision have been reported to occur at 1.2% and 0.8% per year.^23^ To address stability concerns, alternative fixation methods with cementless metal-backed glenoid (MBG) components were developed, but these initial implants were found to have unacceptably high rates of failure secondary to early excessive wear and loosening, screw breakage, and polyethylene dissociation.^1^, ^2^, ^18^, ^19^ Furthermore, the increased thickness of the MBG and associated polyethylene when compared to an all-polyethylene component can lead to an increased incidence of “over-stuffing” of the glenohumeral joint.

The rapidly rising incidence of shoulder arthroplasty is expected to be accompanied by an increased burden of all-cause revision arthroplasty.^14^ When converting to reverse total shoulder arthroplasty, the removal of well-fixed cemented PEG components risks significant bone loss, which substantially increases the complexity of the revision and potentially compromises the revised prosthesis.^26^ Therefore, modular components that can be easily converted from anatomic to reverse configuration without significant bone loss present an attractive option for an index procedure.

To address the issues of glenoid stability and the growing need for easily revisable components, a second-generation of convertible MBG components was recently developed. These implants incorporate highly porous baseplates to support bone ingrowth as well as vitamin E-coated polyethylene liners with improved wear characteristics. Bony integration into the implant confers a potentially more stable and durable construct, and the component modularity obviates the need for baseplate removal when converting from polyethylene glenoid to glenosphere. Short and midterm studies for such devices have shown encouraging results; however, there is a paucity of longer-term data reported in the literature.^12^, ^13^

This study reports on the radiographic and clinical outcomes of the Comprehensive Convertible Glenoid system (Zimmer Biomet, Warsaw, IN, USA) at an average follow-up of 3.8 years. We hypothesize that patients who underwent aTSA with MBG would have significantly improved function compared to their pre-operative state and that the outcomes in this group will be similar to the reported literature of patients who underwent aTSA with traditional polyethylene implants.

## Methods

This is a retrospective review of prospectively collected data investigating the short-term outcomes of aTSA in patients who received an MBG component in conjunction with their total shoulder arthroplasty. The investigation was performed at a single institution, and the surgeries were performed by 1 of 4 sports or shoulder fellowship-trained surgeons between 2016 and 2020. The institutional review board approved the study (2019-1819). Inclusion criteria included patients undergoing primary aTSA for osteoarthritis with a concern for early polyethylene failure. These criteria included significant retroversion (>20°), posterior subluxation (>80%), cystic change in the glenoid, and medialization of the glenohumeral joint line where cement extravasation would occur. A minimum of 2-year follow-up data were also required (Fig. 1). The exclusion criteria included prior open or arthroscopic surgery, prior proximal humerus fracture, revision shoulder arthroplasty, and inadequate follow-up data. Patients were identified for inclusion after a review of our institutional arthroplasty registry and electronic medical record review.

**Figure 1:**
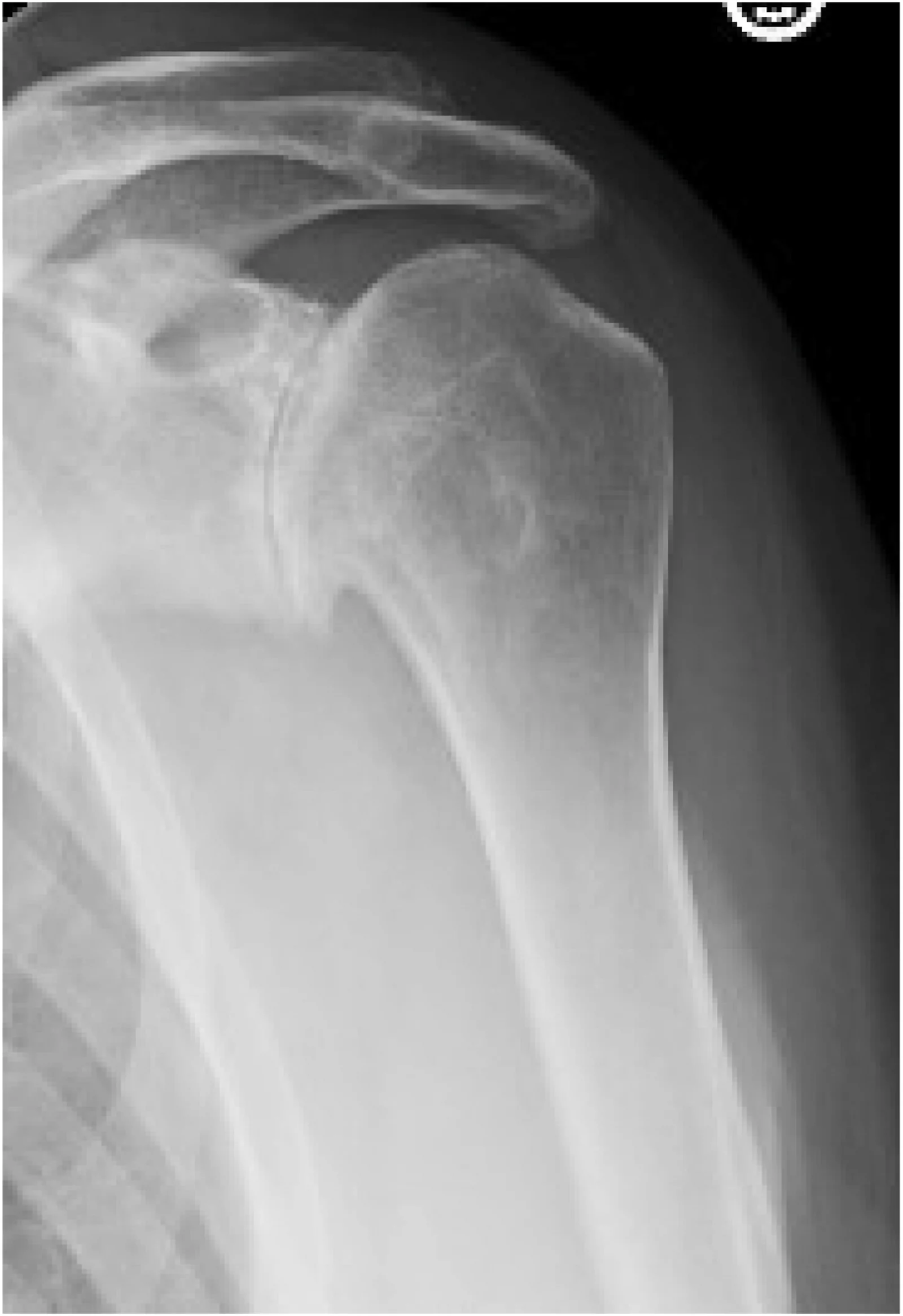
Pre-operative internal rotation view x-ray demonstrating advanced glenohumeral osteoarthritis.

Patient demographic information, comorbidities, pre- and post-operative radiographic measurements, surgical factors, and complications were collected. Patient-reported outcome scores (pre-operative and 2-year) were collected from the institution's Shoulder Arthroplasty Registry and included American Shoulder and Elbow Surgeons (ASES) score, Single Assessment Numeric Evaluation (SANE), Shoulder Activity Scale, Patient-Reported Outcomes Measurement Information System, and visual analog scale (VAS). Patient comorbidities were used to determine the American Society of Anesthesiologists classification. Radiographic measurements included pre-operative retroversion, subluxation, lateral-humeral offset, and acromiohumeral interval.

All surgical procedures were performed using a single glenoid implant system: Comprehensive Convertible Glenoid aTSA system (Zimmer Biomet, Warsaw, IN, USA) (Fig. 2). Two surgeons in this study were involved in the implant design and previously received royalties. All of the humeral stems were cementless; however, there was variation in stem length. All aTSAs were performed through a deltopectoral approach. The subscapularis was taken down either via a subscapularis tenotomy or lesser tuberosity osteotomy and was repaired at the conclusion of the operation based on surgeon preferred technique. As there is no available augment in this system, glenoid deformity was addressed via corrective reaming or bone grafting. If bone grafting was performed, it was with autograft from the humeral head. All glenoid components were fixed with screw fixation as per the manufacturer's guidelines. Post-operatively, patients were allowed to engage in immediate passive range of motion, with 140° of forward elevation. Active and passive external rotation was limited in the first 4-6 weeks following surgery to support subscapularis healing. Six weeks post-operatively, active range of motion was started, and sling use was discontinued. Strengthening programs for the rotator cuff, periscapular, and deltoid muscles was begun at post-operative week 10. Rehabilitation protocols were similar across surgeons.

**Figure 2:**
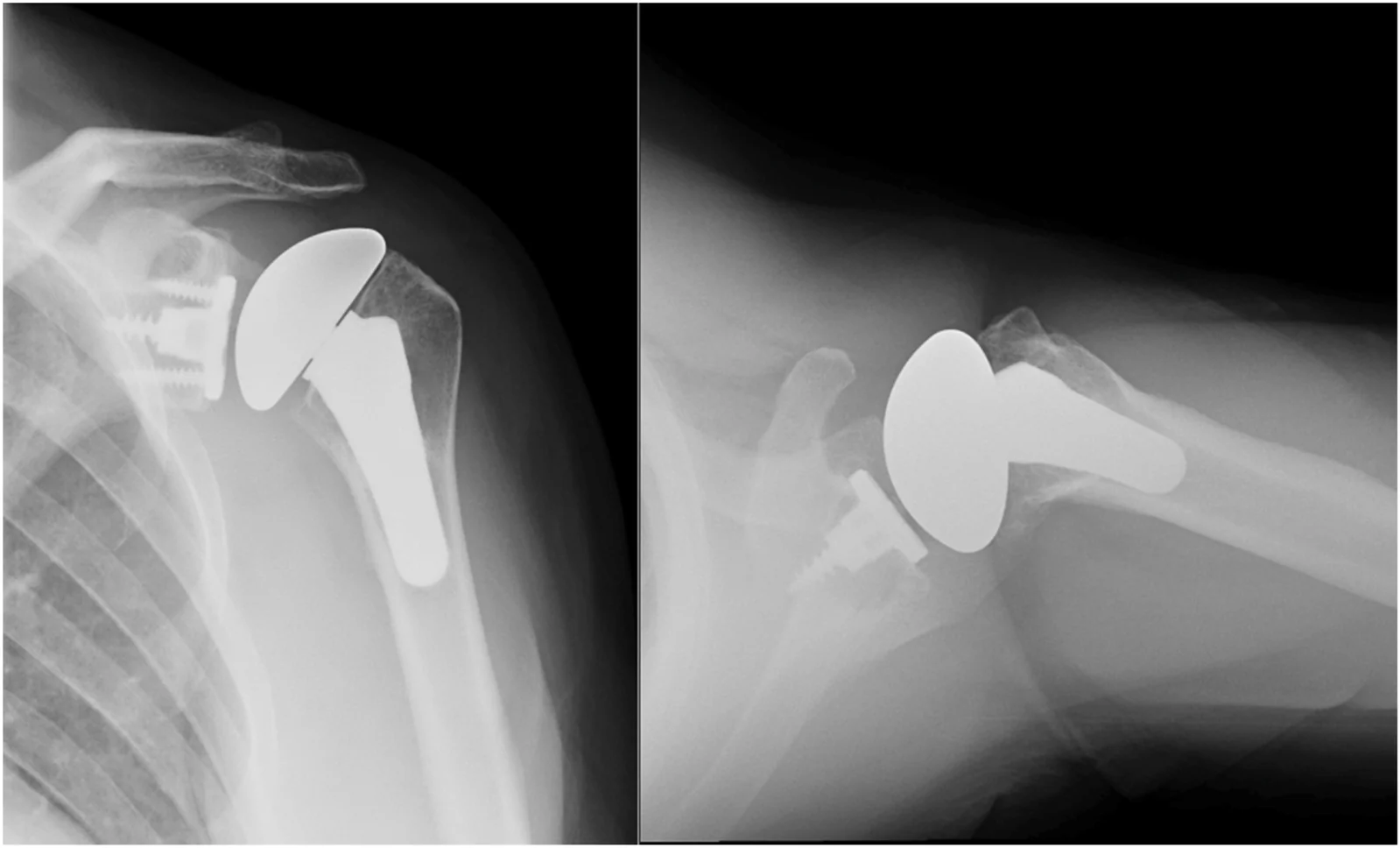
Post-operative Grashey anteroposterior and axillary view x-rays demonstrating anatomic total shoulder arthroplasty with a metal-backed glenoid.

Patients were evaluated pre-operatively, at 1 year post-operatively, and at the final follow-up visit, which varied between 2 and 5 years post-operatively. A thorough physical examination was conducted at each time point, which included passive range of motion, strength in forward flexion, and strength in external rotation with the arm positioned at the side. Range of motion measurements were performed using a goniometer, and strength testing was graded on the standard 0-5 scale. All evaluations were conducted by the operative orthopedic surgeon or a shoulder and elbow fellow.

Statistical analysis was performed using IBM SPSS Statistics 20 software (IBM Corporation, Armonk, NY, USA). The Student’s *t*-test and analysis of variance were utilized to assess differences in outcome scores, range of motion, and strength scores when applicable. Chi-square testing was performed for analysis of nominal data. The reported values represent the average ± 1 standard deviation for each group, and a significance level of *P* < .05 was considered statistically significant.

## Results

Twenty-eight patients met the inclusion criteria, and their demographic and pre-operative radiographic data are summarized in Table I. The primary indication for use of an MBG was in younger male patients with Walch type B glenoids (22 patients); however, additional indications included a cement allergy (1 patient), cystic change in the glenoid compromising cement fixation (1 patient), and significant medialization and glenoid bone loss (4 patients). The cohort included 21 males and 7 females, with an average age of 58 years. The average duration from surgery to final follow-up was 3.8 years. Five patients underwent glenoid bone grafting to improve glenoid retroversion, while the remaining patients either received corrective reaming or in situ glenoid placement. The decision to perform bone grafting was in patients with significant retroversion measuring greater than 20°, with the goal of restoring retroversion to less than 15°.

**Table I:** Patient demographics.

Characteristic	Cohort (n = 28)
Age, yr	58.0 (6.5)
Affected arm	
Left	16
Right	12
Sex	
Male	21
Female	7
BMI	28.3 (4.7)
ASA	2.0 (0.3)
Follow-up, yr	3.8 (1.3)
Walch grade	
A1	4
A2	1
B1	8
B2	11
B3	3
C	1

*ASA,* American Society of Anesthesiologists; *BMI,* body mass index.

The average pre-operative retroversion measured 15.2° (3°-28°) and the pre-operative subluxation measured 70.2% (60%-88%). Post-operatively, these values measured 12.2° (4°-24°) (*P* = .05) and 57.3% (44%-85%) (*P* < .001), respectively.

Data regarding range of motion, strength, and PROMs can be found in Table II. Range of motion statistically improved in all measurements with forward elevation improved from 129° to 166° (*P* < .001), external rotation at the side improved from 34° to 55° (*P* < .001), and internal rotation improved from L5 to T12 (*P* < .001). There was no statistical improvement in supraspinatus (*P* = .115), infraspinatus (*P* = .261), and subscapularis strength (*P* = .082).

**Table II:** Pre- and post-op values.

Range of motion	Pre-op	Post-op	*P* value
Forward elevation (°)	129 (28.8)	166 (12.2)	<.001∗
External rotation (°)	34 (19.0)	55 (12.2)	<.001∗
Internal rotation (level)	L5 (3.1)	T12 (3.6)	<.001∗
Strength (0-5)			
Supraspinatus	4.9 (0.3)	5.0 (0.2)	.115
Infraspinatus	4.9 (0.2)	5.0 (0.2)	.261
Subscapularis	5.0 (0.2)	4.8 (0.4)	.082
Patient-reported outcome measures			
ASES	39.2 (23.3)	86.6 (19.0)	<.001∗
SANE	28.8 (23.3)	79.0 (21.2)	<.001∗
SAS	10.0 (4.5)	13.0 (4.1)	.008∗
PROMIS	33.1 (7.6)	50.9 (10.6)	<.001∗
VAS	5.4 (3.0)	2.6 (3.1)	<.001∗
Radiographic measurements			
Glenoid retroversion (°)	15.2 (7.7)	12.2 (5.5)	.053
Humeral head subluxation (%)	70.3 (9.1)	57.3 (9.3)	<.001∗

*SAS*, Shoulder Activity Scale; *ASES*, American Shoulder and Elbow Surgeons; *SANE*, Single Assessment Numeric Evaluation; *PROMIS*, Patient-Reported Outcomes Measurement Information System; *VAS*, visual analog scale.

∗ Denoted Significant values.

All of the recorded PROMs showed significant improvement from pre-operatively to final follow-up. The ASES score improved 47.4 points on average (*P* < .001, substantial clinical benefit [SCB] 31.5), while the SANE score increased from 28.8 to 79 (*P* < .001, minimal clinically important difference 14.9). The Shoulder Activity Scale demonstrates a more modest improvement from 10.0 to 13.0 (*P* = .008). The upper-extremity Patient-Reported Outcomes Measurement Information System score increased 17.8 (*P* < .001, SCB 10.0) from pre-operative to final follow-up visits, while VAS decreased from 5.2 to 2.6 (*P* < .001, SCB 3.2).

There were no immediate post-operative complications. At final follow-up, 3 patients underwent conversion to reverse shoulder arthroplasty for progressive posterior subluxation of the humeral head (1 patient, 4 years post-op) or subscapularis insufficiency (2 patients, 3 years post-op), with retention of the MBG and humeral components (Fig. 3). The patient with posterior subluxation demonstrated dynamic posterior instability on physical examination and joint-space narrowing on radiograph. The decision to revise to rTSA was made in advance of the catastrophic failure of the implant. At revision, the polyethylene showed posterior wear. The revisions for subscapularis insufficiency did not demonstrate polyethylene wear at the time of surgery. At 1-year revision follow-up, these patients averaged 140° of forward elevation, 40° of external fixation, and internal rotation to the sacrum. Their ASES scores improved from 27.5 pre-operatively to 80 post-operatively. One additional patient had subscapularis insufficiency with sufficient function and elected to defer further surgical intervention. There were no instances of glenoid or humeral component loosening, polyethylene dissociation, or periprosthetic fracture.

**Figure 3:**
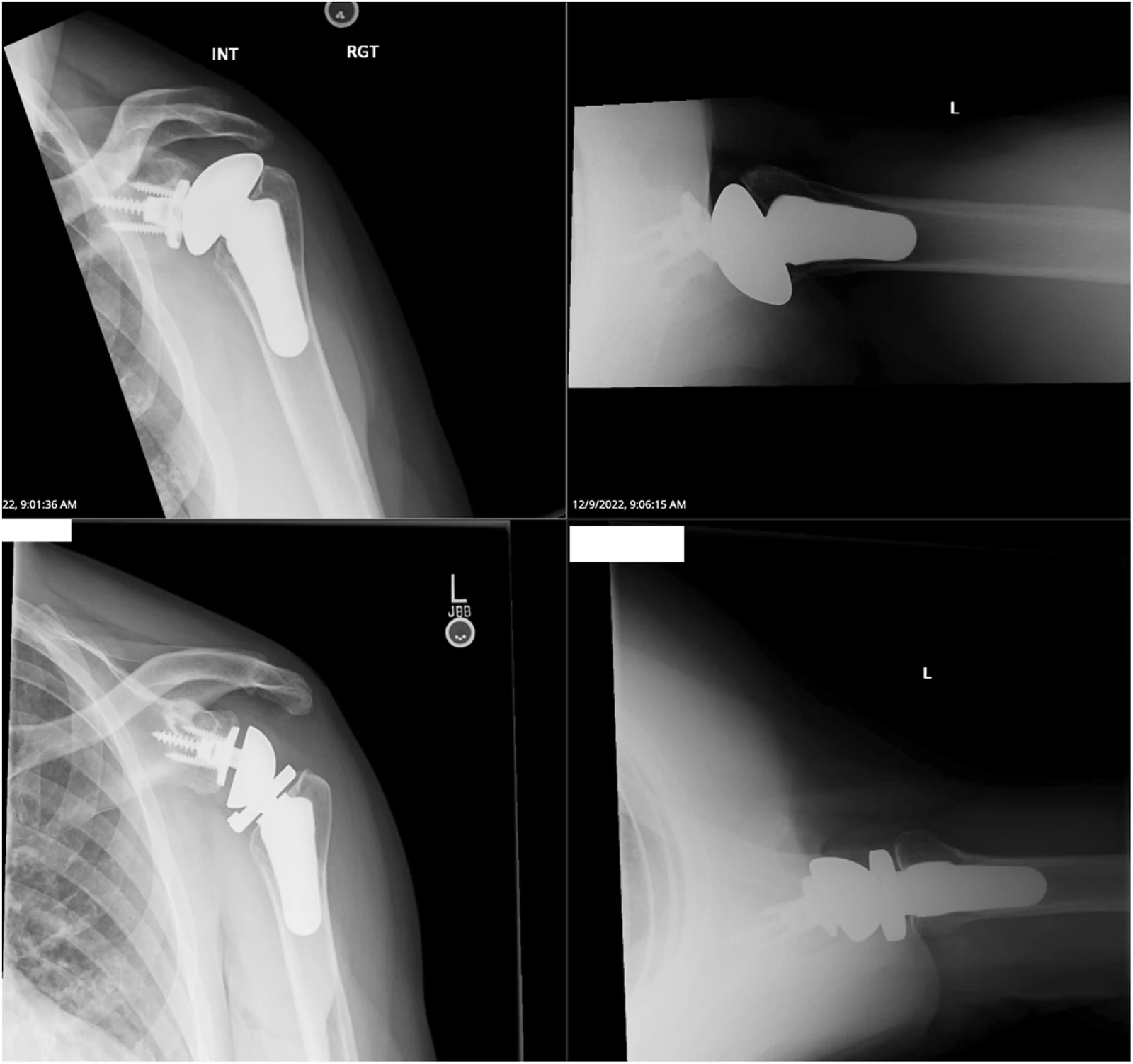
Patient withpost-operative posterior subluxation of humeral head converted to reverse shoulder arthroplasty with stem and baseplate retention. *INT,* internal rotation; *RGT,* right shoulder.

## Discussion

As the incidence of total shoulder arthroplasty increases, there is a need for maximal longevity in the primary setting and minimal morbidity in the revision setting.^6^ Although cemented all-polyethylene glenoid (PEG) components have been considered the gold standard for patients suffering from primary osteoarthritis, many patients still experience glenoid component loosening, with 20% of patients requiring a revision procedure by their 15th year following surgery.^13^

MBGs were introduced in 2003 to address the issue of aseptic loosening by promoting bony ingrowth into the implant.^16^ Additionally, MBGs are designed to solve complications involving glenoid deformities, including significant retroversion and humeral head subluxation, particularly in younger patients. These deformities can lead to premature implant failure of traditional shoulder arthroplasty implants due to the “rocking horse phenomenon,” where eccentric loading of the glenoid component causes loosening over time.^5^ A commonly accepted solution to this problem is bone grafting with an aTSA.^24^ This technique is ideal for younger patients because their motion is preserved. The MBG design aims to address the rocking horse phenomenon by enabling graft fixation to the glenoid component, thereby reducing the potential for loosening.^5^

The first generation of MBG components introduced in 2003 was found to have high rates of failure due to fractures at the keel-glenoid face junction, implant loosening, and polyethylene wear.^13^, ^16^ These original designs included an increase in the total thickness of the glenoid component between the polyethylene and metal tray. This increased the risk of overstuffing the glenohumeral joint if not accounted for, which may contribute to early subscapularis failures, increased polyethylene wear, subsequent rotator cuff insufficiency, and suboptimal outcomes. Initial MBG designs showed a revision rate 3x higher than traditional cemented PEG components.^13^ Additionally, neither the first-generation MBG components nor the cemented PEG components were well suited to revision. If a patient developed rotator cuff insufficiency following aTSA and was indicated for revision to a reverse shoulder arthroplasty, extraction of the implant might lead to severe glenoid bone loss, complicating the revision process.^13^ The increased incidence of initial MBG aTSA failure combined with poor revision prognosis necessitated a new MBG design.

In 2009, a second-generation MBG design was introduced using porous tantalum in the backside of the implant (Zimmer Biomet, Warsaw, IN, USA).^17^ To address component loosening, the new implant was designed to increase fixation to the glenoid bone contact area with a porous-coated baseplate. The baseplate's success also depends on screw fixation, similar to the fixation used in the reverse shoulder arthroplasty implant from Zimmer Biomet. Additionally, the liner was redesigned to reduce overall thickness and include vitamin E1 to improve durability against polyethylene wear.^13^ This second-generation MBG component addressed issues related to revision by incorporating convertible and modular parts that allowed for a less complicated conversion to reverse shoulder arthroplasty. Specifically, a removable polyethylene liner was introduced that could be converted to a glenosphere without removing the baseplate.^13^

These changes in the second-generation MBG glenoid resulted in improved outcomes compared to the first-generation implant. Several studies have reported short-term and midterm results similar to those found in this study. Chin et al^3^ compared outcomes in 46 s-generation MBG patients with those in 47 cemented all-polyethylene patients at 2- and 5-year time points. There were no significant differences in post-operative outcomes, including ASES and VAS scores, between the 2 groups. Additionally, a study conducted by DiPasquale et al^4^ followed 26 patients and showed no evidence of implant loosening or hardware failure of any kind at >2 years follow-up. Murphy et al^21^ followed 39 patients who underwent aTSA with a second-generation MBG implant and found a 100% survivorship rate at a mean follow-up of 7.2 years. Patients had an average forward elevation of 153° ± 22° and an average external rotation of 53° ± 12°.^21^ The average ASES score was 86.8 ± 19.0, and the average VAS score was 1.3 ± 2.4, reflecting similar patient-reported outcome measures and ROM values to the ones reported in this study.^21^

When compared to the short-term follow-up results from PEG implants, our reported outcomes are comparable and also reflect an increased mean forward flexion of 166°. Moulton et al^20^ collected results from 47 shoulders that underwent aTSA with a cemented all-polyethylene pegged glenoid and reported a mean forward elevation of 131° and mean ASES, SANE, and VAS values of 87.0, 89.1, and 0.9, respectively. These improvements exceed the published substantial clinical benefit for shoulder arthroplasty, representing not only statistical but also a clinically relevant improvement following aTSA with a metal-back glenoid design.

Haque et al^9^ report midterm outcomes of 108 shoulders that underwent an aTSA with a cemented all-polyethylene implant. These results are less favorable than those reported here for MBG patients, with a mean forward elevation of 111° and a mean VAS of 1.8 at 5.1 years of follow-up.^9^ Additionally, the reported post-operative range of motion values in this study are greater than those typically seen in reverse shoulder arthroplasty cases, especially when considering internal rotation.^11^ This may indicate that this approach could be more beneficial for younger patients seeking to return to high activity levels following surgery.

Studies have raised concerns regarding the use of MBG implants for anatomic shoulder arthroplasty.^8^, ^27^ There are several factors associated with premature polyethylene wear in MBGs. The most prominent risk factor is specifically related to the size of the implant. The MBG components are thicker than the all-polyethylene components because they must include enough metal for proper fixation to the bone as well as sufficient polyethylene to allow for wear. The increased thickness of the glenoid component can result in increased tensioning of the subscapularis and over-lateralization of the articular surface.^11^ The most prominent downstream effects of these issues are subsequent subscapularis or supraspinatus failure with the potential need for conversion to reverse shoulder arthroplasty.^23^

One noteworthy finding in this study is the conversion of 3 patients to reverse shoulder arthroplasty with retention of the glenoid baseplate and humeral stem. The convertibility of the glenoid and humeral components can significantly reduce the morbidity to the patient, technical difficulty, and operative time of revision surgery. In patients with an adequate baseplate inclination, the use of a variably eccentric glenosphere (Versadial; Zimmer Biomet, Warsaw, IN, USA) can provide a customizable inferior offset, thereby minimizing the risk of subsequent impingement or scapular notching. The 3 revision patients demonstrated good clinical results without surgical complications at 1 year post-operatively.

Given the relatively small sample size of our study, we acknowledge the need for future studies with larger samples. Additionally, all patients are from a single institution and were operated on by 4 surgeons using a single implant system, which limited the generalizability of the study's findings. Expanding the study to include multiple institutions with an increased number of patients and multiple MBG designs would improve the ability to draw clinically impactful conclusions from the study. Here, we report on short-term outcomes but acknowledge the need for midterm to long-term follow-up of this patient population to provide a complete picture of implant success rates.

## Conclusion

The use of the Comprehensive Convertible Glenoid system in complex total shoulder arthroplasty results in excellent improvement in radiographic and clinical outcomes in short-term follow-up. Additionally, the system provides a method of convertibility if needed in conversion to reverse shoulder arthroplasty. The information in this article can be helpful in counseling patients about the short-term results of this procedure. Future studies should focus on long-term outcomes and include larger, multicenter cohorts to validate these findings further and enhance the generalizability of the results.

## Disclaimers:

Funding: No funding was disclosed by the authors.

Conflicts of interest: Joshua S. Dines reports that he serves as a board or committee member of ASES; receives consultant fees from Arthrex, royalties from Arthrex and Conmed Linvatec, research support from Arthrex and HSS, and publishing royalties from Thieme Inc. and Lippincott Williams & Wilkins; and serves on the Board of Directors of Viewfi.

Michael C. Fu reports that he serves on the Editorial or Governing Board for Arthroscopy and the HSS Journal and receives consultant fees from Stryker.

Samuel A. Taylor reports that he is a Don Joy Orthopedics - Designer and receives honoraria from Don Joy Orthopedics.

David M. Dines reports that he serves as a board or committee member for ASES and AAOS; receives publishing royalties from Thieme Inc., consultant fees from Wright Medical Technology and Zimmer Biomet Inc., and royalties for the design and invention of bimodular, total, and comprehensive shoulder replacement systems; and serves on the Editorial Board for Springer.

Russell F. Warren reports that he receives royalties from Arthrex, Inc, and has Stryker and Agelity Biomechanics ownership interest.

Lawrence V. Gulotta reports that he has a Responsive Arthroscopy ownership interest and an ownership interest in Imagen Inc., serves on the Smith & Nephew Speakers’ Bureau, serves as a consultant and on the speakers’ bureau for Zimmer-Biomet, Inc., and serves on the Editorial or Governing Board of the HSS Journal.

Any additional authors, their immediate families, and any research foundations with which they are affiliated have not received any financial payments or other benefits from any commercial entity related to the subject of this article.

## Ethics declaration

Written informed consent to take part in the study and to publish the article has been obtained from all participants or their legal representatives. The privacy rights of participants have been observed.

This study was performed in compliance with relevant laws, regulatory frameworks, and guidelines where the research took place.
